# Bis[4-amino-*N*-(pyrimidin-2-yl-κ*N*)benzene­sulfonamidato-κ*N*](4,4′-di­methyl-2,2′-bipyridine-κ^2^ *N*,*N*′)cadmium dimethyl­formamide disolvate

**DOI:** 10.1107/S1600536812004412

**Published:** 2012-02-10

**Authors:** G. M. Golzar Hossain, A. J. Amoroso

**Affiliations:** a Department of Chemistry, University of Dhaka, Dhaka 1000, Bangladesh; b School of Chemistry, Cardiff University, Cardiff CF10 3AT, Wales

## Abstract

In the title compound, [Cd(C_10_H_9_N_4_O_2_S)_2_(C_12_H_12_N_2_)]·2C_3_H_7_NO, the Cd^II^ ion lies on a twofold rotation axis, is six-coordinated by N atoms, and displays a trigonal–prismatic geometry arising from the two sulfadiazinate ligands and one 4,4′-dimethyl-2,2′-bipyridine ligand. Both ligands are bidentate and coordinate *via* their N atoms. The O and carbonyl C atoms of the dimethyl­formamide mol­ecule show disorder and were modelled with two different orientations and with site occupancies of 0.584 (10):0.416 (10). The geometry around the sulfadiazine S atom is distorted tetra­hedral. The crystal structure involves N—H⋯O hydrogen bonds which link mol­ecules into a three-dimensional network. Weak C—H⋯O hydrogen bonds are also observed.

## Related literature

For the comparison of the N—H bond distance of the terminal amine group and the C—S—N—C torsion angle, see: Heren *et al.* (2006 ▶); Hossain & Amoroso (2007 ▶); Hossain (2011 ▶). For the hydrogen bonds of sulfadiazinate anions, see: Paşaoğlu *et al.* (2008 ▶). For the comparison of the dihedral angle between the aromatic rings of the anion, see: Hossain & Amoroso (2007 ▶); Hossain (2011 ▶). For the comparison of Cd—N bond distances, see: Kalateh *et al.* (2010 ▶); Hossain (2011 ▶).
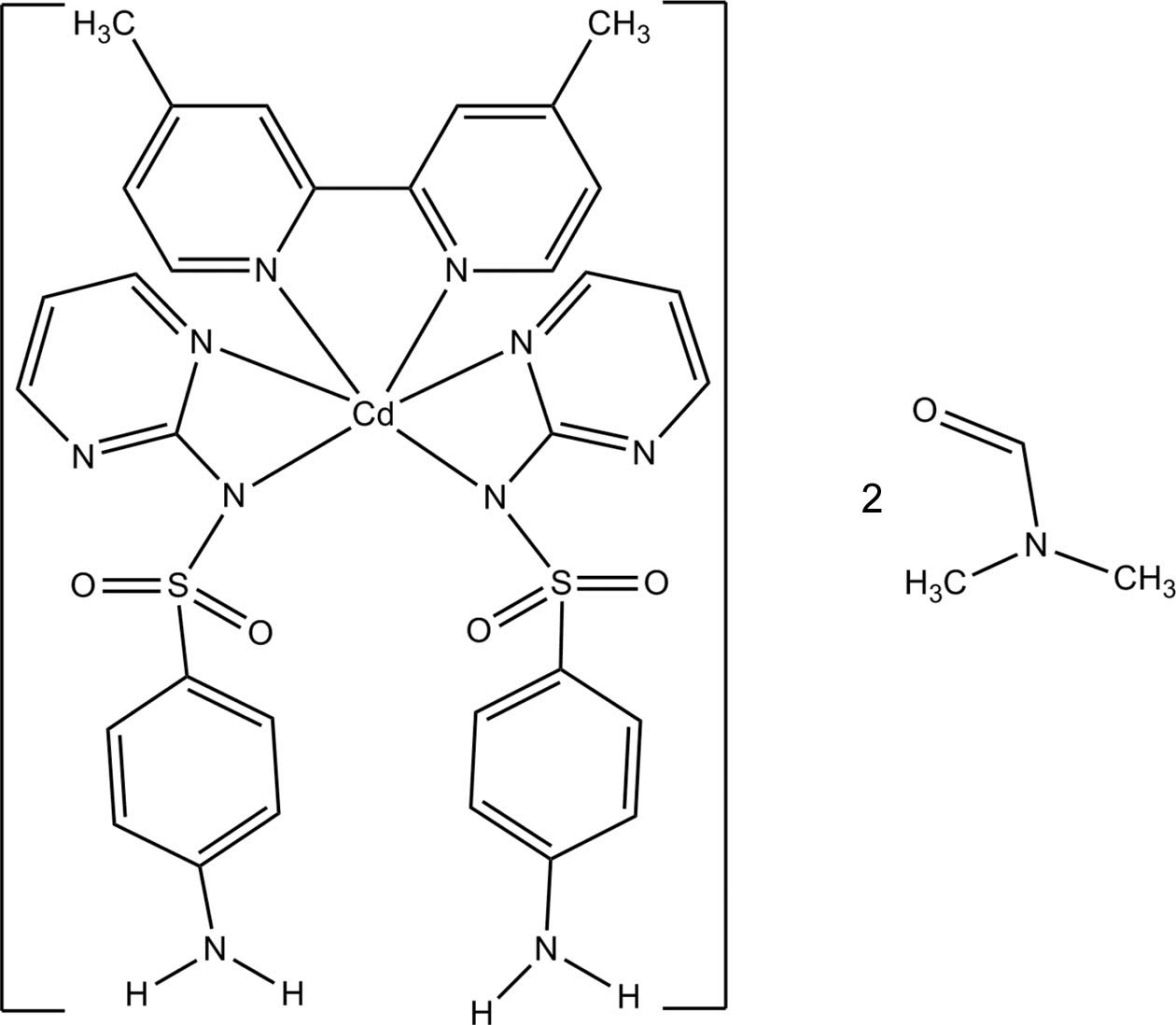


## Experimental

### 

#### Crystal data

[Cd(C_10_H_9_N_4_O_2_S)_2_(C_12_H_12_N_2_)]·2C_3_H_7_NO
*M* *_r_* = 941.37
Monoclinic, 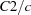

*a* = 17.4428 (4) Å
*b* = 16.2753 (4) Å
*c* = 16.3873 (4) Å
β = 118.3334 (11)°
*V* = 4094.81 (17) Å^3^
*Z* = 4
Mo *K*α radiation
μ = 0.70 mm^−1^
*T* = 150 K
0.20 × 0.20 × 0.18 mm

#### Data collection

Nonius KappaCCD diffractometer
Absorption correction: multi-scan (Blessing, 1995 ▶) *T*_min_ = 0.873, *T*_max_ = 0.885
18995 measured reflections
4685 independent reflections
3855 reflections with *I* > 2σ(*I*)
*R*_int_ = 0.070

#### Refinement

*R*[*F* ^2^ > 2σ(*F* ^2^)] = 0.038
*wR*(*F* ^2^) = 0.093
*S* = 1.04
4685 reflections
297 parameters
30 restraints
H atoms treated by a mixture of independent and constrained refinement
Δρ_max_ = 0.55 e Å^−3^
Δρ_min_ = −0.59 e Å^−3^

### 

Data collection: *DENZO* (Otwinowski & Minor, 1997 ▶) and *COLLECT* (Hooft, 1998 ▶); cell refinement: *DENZO* and *COLLECT*; data reduction: *DENZO* and *COLLECT*; program(s) used to solve structure: *SHELXS97* (Sheldrick, 2008 ▶); program(s) used to refine structure: *SHELXL97* (Sheldrick, 2008 ▶); molecular graphics: *ORTEP-3 for Windows* (Farrugia, 1997 ▶); software used to prepare material for publication: *WinGX* (Farrugia, 1999 ▶).

## Supplementary Material

Crystal structure: contains datablock(s) I, global. DOI: 10.1107/S1600536812004412/rn2090sup1.cif

Structure factors: contains datablock(s) I. DOI: 10.1107/S1600536812004412/rn2090Isup2.hkl

Additional supplementary materials:  crystallographic information; 3D view; checkCIF report

## Figures and Tables

**Table:** 

Cd1—N1	2.312 (2)
Cd1—N11	2.251 (2)
Cd1—N12	2.505 (2)
N14—C18	1.360 (3)

**Table:** 

N11^i^—Cd1—N11	116.97 (11)
N11—Cd1—N1^i^	128.97 (8)
N11—Cd1—N1	102.88 (8)
N1^i^—Cd1—N1	70.87 (11)
N11—Cd1—N12^i^	95.54 (7)
N1—Cd1—N12^i^	91.26 (7)
N11—Cd1—N12	56.10 (7)
N1—Cd1—N12	134.18 (8)
N12^i^—Cd1—N12	127.65 (9)
N13—C11—N12	125.7 (2)

Symmetry code: (i) 
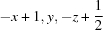
.

**Table 2:** Hydrogen-bond geometry (Å, °)

*D*—H⋯*A*	*D*—H	H⋯*A*	*D*⋯*A*	*D*—H⋯*A*
N14—H14*B*⋯O1*D*^ii^	0.95 (1)	2.10 (1)	3.034 (7)	169 (3)
N14—H14*B*⋯O1^ii^	0.95 (1)	1.99 (2)	2.853 (5)	151 (3)
N14—H14*A*⋯O11^iii^	0.95 (1)	2.00 (1)	2.950 (3)	176 (3)
C12—H12⋯O12^iv^	0.95	2.48	3.393 (3)	162
C6—H6*C*⋯O1^v^	0.98	2.58	3.487 (5)	154
C6—H6*A*⋯N13^vi^	0.98	2.63	3.558 (4)	159
C8—H8*A*⋯O11^vii^	0.98	2.55	3.497 (4)	161

Symmetry codes: (ii) 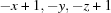; (iii) 
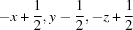
; (iv) 
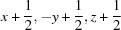
; (v) 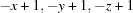; (vi) 
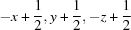
; (vii) 
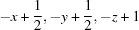
.

**Fig. 1.**
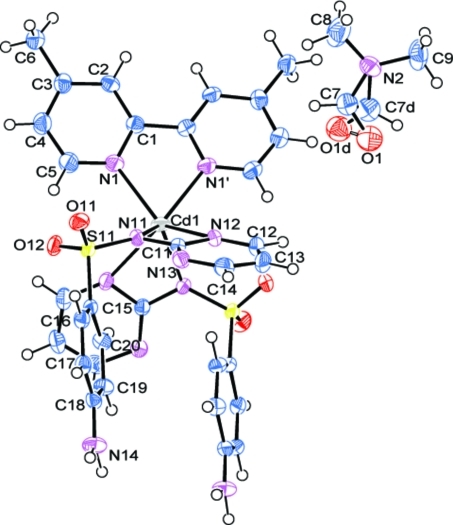
The molecular structure of the title compound (I), with atom labels and 50% probability displacement ellipsoids for non-H atoms. The disordered atoms are linked by dashed lines.

**Fig. 2.**
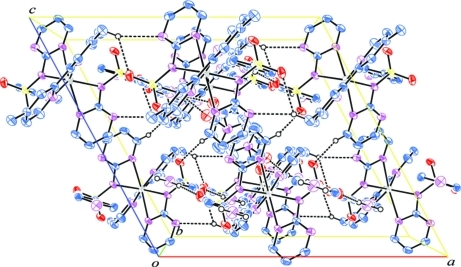
The packing of (I), viewed down the b-axis, showing one layer of molecules connected by N—H···O hydrogen bonds (dashed lines).

## supplementary crystallographic information

### Comment

The cadmium complex is six coordinate and shows trigonal prismatic rather than the octahedral structure as the *cis* and *trans* angles around the cadmium centre deviate considerably from the ideal octahedron [*cis* angle of 56.10 (7) *cf.* 90° and *trans* angle of 128.97 (8) *cf.* 180°]. The bond angles around the S atom correspond to a distorted tetrahedral geometry.

The bond distance C18–N14 of 1.359 (3)Å is comparable with the value of 1.366 (5)Å (Hossain, 2011). The torsion angle C15–S11–N11–C11 of 53.5 (2)° is less than the value of 66.1 (3)° and the dihedral angle between the aromatic rings of the anion of 76.60 (8)° is also smaller than the value of 88.65 (12)° in the sulfadiazinate anion (Hossain, 2011) because the large 4,4'-dimethyl-2,2'-bipyridine (dmbpy) ligand is attached to the Cd ion in the complex. Due to the presence of the larger dmbpy molecule the torsion and dihedral angles are reduced from the latter one where small dmf molecules are attached with the metal centre. In the title complex, (I), the O and formido C atoms of the solvated dimethylformamide show disorder and were modeled as two different orientations with site occupancies of 0.584 (10):0.416 (10).

Cd–N1(dmbpy) bond distance of 2.312 (2)Å is consistent with those for the reported dmbpy-Cd(II) complex, (Cd–N 2.366 (5) and 2.326 (4) Å)(Kalateh *et al.*, 2010). Cd–N11(sulfonamido) bond distance of 2.252 (2)Å is relatively short (Hossain, 2011) and Cd—N12(pyrimido) with the value of 2.505 (2)Å is the longest bond in the complex.

The packing of (1) (Fig. 2) is stabilized by intermolecular N—H···O hydrogen bonds (Table 2) between the sdz anions (Paşaoğlu, *et al.*, 2008) and dimethylformamide molecules.

### Experimental

The sodium salt of sulfadiazine (Nasdz, 0.5446 g, 2 mmol) was dissolved in hot methanol (50 ml) and a methanol solution (10 ml) of (CH_3_COO)_2_Cd.2H_2_O (0.26647 g, 1 mmol) was added slowly with constant stirring on a hot plate. A white precipitate was formed and the mixture was stirred for a further 2 h. The precipitate was filtered off and dried over silica gel; it was then dissolved in dimethylsulfoxide solution (50 ml), and 4,4'-dimethyl-2,2'-bipyridine (0.1841 g, 1 mmol) was added, stirred for 10 min., filtered and left for crystallization. A week later, white block-shaped crystals of (1) were filtered off and dried over silica gel.

### Refinement

The O and formido C atoms of dimethylformamide show disorder and were modeled with two different orientations and site occupancies of 0.584 (10):0.416 (10). The H atoms were positioned geometrically and refined using a riding model [except terminal amino group N(14) which were located from the difference map and refined freely with the N—H distances of 0.948 (3) Å], with C—H = 0.95–0.98 Å and with *U*_iso_(H) = 1.2 (1.5 for methyl groups) times *U*_eq_(C).

### Crystal data

**Table:**

[Cd(C_10_H_9_N_4_O_2_S)_2_(C_12_H_12_N_2_)]·2C_3_H_7_NO	*F*(000) = 1936
*M**_r_* = 941.37	*D*_x_ = 1.527 Mg m^−^^3^
Monoclinic, *C*2/*c*	Mo *K*α radiation, λ = 0.71073 Å
Hall symbol: -C 2yc	Cell parameters from 4685 reflections
*a* = 17.4428 (4) Å	θ = 2.9–27.5°
*b* = 16.2753 (4) Å	µ = 0.70 mm^−^^1^
*c* = 16.3873 (4) Å	*T* = 150 K
β = 118.3334 (11)°	Block, white
*V* = 4094.81 (17) Å^3^	0.20 × 0.20 × 0.18 mm
*Z* = 4	

### Data collection

**Table:**

Nonius KappaCCD diffractometer	4685 independent reflections
Radiation source: fine-focus sealed tube	3855 reflections with *I* > 2σ(*I*)
Graphite monochromator	*R*_int_ = 0.070
ω scans	θ_max_ = 27.5°, θ_min_ = 3.4°
Absorption correction: multi-scan (Blessing, 1995)	*h* = −22→22
*T*_min_ = 0.873, *T*_max_ = 0.885	*k* = −20→21
18995 measured reflections	*l* = −21→21

### Refinement

**Table:**

Refinement on *F*^2^	Primary atom site location: structure-invariant direct methods
Least-squares matrix: full	Secondary atom site location: difference Fourier map
*R*[*F*^2^ > 2σ(*F*^2^)] = 0.038	Hydrogen site location: inferred from neighbouring sites
*wR*(*F*^2^) = 0.093	H atoms treated by a mixture of independent and constrained refinement
*S* = 1.04	*w* = 1/[σ^2^(*F*_o_^2^) + (0.0369*P*)^2^ + 6.0396*P*] where *P* = (*F*_o_^2^ + 2*F*_c_^2^)/3
4685 reflections	(Δ/σ)_max_ < 0.001
297 parameters	Δρ_max_ = 0.55 e Å^−^^3^
30 restraints	Δρ_min_ = −0.59 e Å^−^^3^

### Special details

**Table:**

Geometry. All e.s.d.'s (except the e.s.d. in the dihedral angle between two l.s. planes) are estimated using the full covariance matrix. The cell e.s.d.'s are taken into account individually in the estimation of e.s.d.'s in distances, angles and torsion angles; correlations between e.s.d.'s in cell parameters are only used when they are defined by crystal symmetry. An approximate (isotropic) treatment of cell e.s.d.'s is used for estimating e.s.d.'s involving l.s. planes.
Refinement. Refinement of *F*^2^ against ALL reflections. The weighted *R*-factor *wR* and goodness of fit *S* are based on *F*^2^, conventional *R*-factors *R* are based on *F*, with *F* set to zero for negative *F*^2^. The threshold expression of *F*^2^ > σ(*F*^2^) is used only for calculating *R*-factors(gt) *etc*. and is not relevant to the choice of reflections for refinement. *R*-factors based on *F*^2^ are statistically about twice as large as those based on *F*, and *R*- factors based on ALL data will be even larger.

### Fractional atomic coordinates and isotropic or equivalent isotropic displacement parameters (Å^2^)

**Table:**

	*x*	*y*	*z*	*U*_iso_*/*U*_eq_	Occ. (<1)
Cd1	0.5000	0.285613 (15)	0.2500	0.02672 (10)	
S11	0.30729 (4)	0.17732 (4)	0.20921 (4)	0.02384 (15)	
O11	0.24693 (12)	0.19449 (12)	0.24412 (14)	0.0332 (4)	
O12	0.28358 (12)	0.20915 (11)	0.11776 (12)	0.0311 (4)	
N11	0.40303 (13)	0.21330 (12)	0.27493 (14)	0.0242 (5)	
N12	0.53497 (14)	0.21773 (13)	0.40087 (14)	0.0253 (5)	
N13	0.42490 (14)	0.13244 (14)	0.40597 (14)	0.0282 (5)	
N14	0.34151 (18)	−0.18335 (14)	0.19655 (18)	0.0384 (6)	
C11	0.45350 (16)	0.18555 (15)	0.36375 (17)	0.0231 (5)	
C12	0.59239 (18)	0.19244 (18)	0.48605 (18)	0.0330 (6)	
H12	0.6500	0.2136	0.5142	0.040*	
C13	0.56952 (19)	0.13622 (19)	0.53386 (19)	0.0368 (7)	
H13	0.6105	0.1171	0.5936	0.044*	
C14	0.48492 (19)	0.10921 (18)	0.49108 (18)	0.0331 (6)	
H14	0.4677	0.0717	0.5238	0.040*	
C15	0.31751 (15)	0.07013 (15)	0.20727 (16)	0.0222 (5)	
C16	0.26797 (16)	0.01885 (16)	0.23138 (17)	0.0262 (5)	
H16	0.2286	0.0417	0.2500	0.031*	
C17	0.27577 (16)	−0.06534 (16)	0.22831 (17)	0.0268 (6)	
H17	0.2412	−0.1000	0.2444	0.032*	
C18	0.33400 (17)	−0.10060 (16)	0.20182 (16)	0.0264 (6)	
C19	0.38526 (18)	−0.04727 (16)	0.17978 (18)	0.0290 (6)	
H19	0.4262	−0.0696	0.1630	0.035*	
C20	0.37690 (17)	0.03677 (16)	0.18217 (17)	0.0259 (5)	
H20	0.4116	0.0719	0.1667	0.031*	
N1	0.42193 (15)	0.40137 (14)	0.17391 (15)	0.0305 (5)	
C1	0.45450 (17)	0.47574 (16)	0.21050 (18)	0.0266 (6)	
C2	0.40492 (17)	0.54691 (16)	0.17667 (18)	0.0270 (6)	
H2	0.4285	0.5985	0.2043	0.032*	
C3	0.32125 (17)	0.54268 (18)	0.10280 (18)	0.0298 (6)	
C4	0.29046 (19)	0.46709 (19)	0.0646 (2)	0.0375 (7)	
H4	0.2341	0.4620	0.0127	0.045*	
C5	0.34180 (19)	0.39805 (19)	0.1019 (2)	0.0391 (7)	
H5	0.3190	0.3460	0.0750	0.047*	
C6	0.26532 (18)	0.61872 (18)	0.0669 (2)	0.0358 (7)	
H6A	0.2244	0.6211	0.0921	0.054*	
H6B	0.2328	0.6167	−0.0010	0.054*	
H6C	0.3025	0.6676	0.0863	0.054*	
N2	0.52238 (17)	0.33948 (15)	0.96056 (17)	0.0372 (6)	
C8	0.4460 (2)	0.3874 (3)	0.9325 (4)	0.0815 (15)	
H8A	0.3993	0.3653	0.8745	0.122*	
H8B	0.4580	0.4444	0.9228	0.122*	
H8C	0.4280	0.3856	0.9807	0.122*	
C9	0.5952 (2)	0.3585 (2)	1.0476 (2)	0.0591 (10)	
H9A	0.6398	0.3159	1.0638	0.089*	
H9B	0.5766	0.3610	1.0953	0.089*	
H9C	0.6194	0.4118	1.0435	0.089*	
C7	0.5088 (5)	0.2855 (3)	0.8943 (4)	0.0388 (16)	0.584 (10)
H7	0.4531	0.2804	0.8415	0.047*	0.584 (10)
O1	0.5723 (3)	0.2409 (3)	0.9032 (3)	0.0543 (17)	0.584 (10)
C7D	0.5528 (7)	0.2831 (6)	0.9201 (7)	0.049 (2)	0.416 (10)
H7'	0.6071	0.2589	0.9614	0.058*	0.416 (10)
O1D	0.5248 (4)	0.2615 (4)	0.8480 (5)	0.054 (3)	0.416 (10)
H14A	0.3127 (18)	−0.2243 (13)	0.213 (2)	0.044 (9)*	
H14B	0.3791 (19)	−0.2145 (17)	0.182 (3)	0.060 (11)*	

### Atomic displacement parameters (Å^2^)

**Table:**

	*U*^11^	*U*^22^	*U*^33^	*U*^12^	*U*^13^	*U*^23^
Cd1	0.02882 (16)	0.01675 (15)	0.03504 (16)	0.000	0.01553 (12)	0.000
S11	0.0178 (3)	0.0207 (3)	0.0262 (3)	0.0004 (2)	0.0049 (2)	−0.0001 (2)
O11	0.0238 (9)	0.0312 (10)	0.0451 (11)	0.0031 (8)	0.0167 (8)	−0.0055 (9)
O12	0.0249 (9)	0.0288 (10)	0.0266 (9)	0.0017 (8)	0.0016 (7)	0.0067 (8)
N11	0.0202 (10)	0.0206 (11)	0.0244 (10)	−0.0030 (8)	0.0047 (8)	−0.0018 (9)
N12	0.0205 (10)	0.0239 (11)	0.0252 (10)	−0.0010 (9)	0.0057 (8)	−0.0039 (9)
N13	0.0298 (11)	0.0270 (12)	0.0243 (11)	−0.0017 (9)	0.0101 (9)	−0.0010 (9)
N14	0.0512 (16)	0.0190 (12)	0.0487 (15)	−0.0013 (11)	0.0268 (13)	0.0009 (11)
C11	0.0221 (12)	0.0189 (12)	0.0240 (12)	0.0014 (10)	0.0074 (10)	−0.0032 (10)
C12	0.0257 (13)	0.0362 (16)	0.0269 (13)	0.0034 (12)	0.0043 (11)	−0.0045 (12)
C13	0.0341 (15)	0.0438 (17)	0.0228 (13)	0.0078 (13)	0.0057 (11)	0.0012 (12)
C14	0.0428 (16)	0.0298 (15)	0.0280 (13)	0.0024 (13)	0.0179 (12)	0.0028 (12)
C15	0.0188 (12)	0.0200 (12)	0.0222 (11)	−0.0014 (9)	0.0050 (9)	−0.0004 (10)
C16	0.0197 (12)	0.0293 (14)	0.0265 (12)	−0.0015 (10)	0.0085 (10)	0.0000 (11)
C17	0.0231 (13)	0.0280 (14)	0.0255 (12)	−0.0051 (11)	0.0084 (10)	0.0032 (11)
C18	0.0300 (14)	0.0233 (13)	0.0199 (11)	−0.0032 (11)	0.0068 (10)	0.0011 (10)
C19	0.0341 (15)	0.0267 (14)	0.0309 (13)	0.0002 (12)	0.0191 (12)	−0.0018 (11)
C20	0.0282 (13)	0.0241 (13)	0.0286 (13)	−0.0048 (11)	0.0159 (11)	−0.0008 (11)
N1	0.0304 (12)	0.0213 (11)	0.0362 (12)	−0.0023 (9)	0.0128 (10)	−0.0008 (10)
C1	0.0267 (14)	0.0255 (13)	0.0290 (13)	−0.0021 (11)	0.0143 (11)	−0.0015 (11)
C2	0.0315 (14)	0.0211 (13)	0.0296 (13)	−0.0046 (11)	0.0154 (11)	−0.0025 (11)
C3	0.0259 (13)	0.0370 (16)	0.0270 (13)	0.0012 (12)	0.0129 (11)	0.0004 (12)
C4	0.0290 (15)	0.0374 (17)	0.0379 (16)	−0.0034 (13)	0.0092 (12)	0.0008 (13)
C5	0.0338 (16)	0.0275 (15)	0.0447 (16)	−0.0060 (12)	0.0095 (13)	−0.0008 (13)
C6	0.0289 (14)	0.0339 (16)	0.0377 (15)	0.0005 (12)	0.0103 (12)	−0.0018 (13)
N2	0.0423 (14)	0.0328 (13)	0.0374 (13)	−0.0080 (11)	0.0195 (11)	−0.0039 (11)
C8	0.041 (2)	0.056 (3)	0.106 (4)	−0.0036 (19)	0.002 (2)	0.013 (2)
C9	0.0377 (18)	0.059 (2)	0.054 (2)	−0.0155 (17)	0.0009 (16)	0.0119 (18)
C7	0.0388 (18)	0.0393 (18)	0.0386 (18)	−0.0027 (10)	0.0186 (11)	0.0007 (10)
O1	0.0560 (19)	0.0551 (19)	0.0553 (19)	0.0001 (10)	0.0294 (12)	−0.0005 (9)
C7D	0.049 (3)	0.048 (3)	0.049 (3)	0.0000 (10)	0.0236 (14)	0.0008 (10)
O1D	0.055 (3)	0.055 (3)	0.055 (3)	−0.0006 (10)	0.0273 (15)	−0.0027 (10)

### Geometric parameters (Å, º)

**Table:**

Cd1—N1	2.312 (2)	C19—H19	0.9500
Cd1—N11	2.251 (2)	C20—H20	0.9500
Cd1—N11^i^	2.251 (2)	N1—C5	1.336 (4)
Cd1—N1^i^	2.312 (2)	N1—C1	1.350 (3)
Cd1—N12^i^	2.505 (2)	C1—C2	1.394 (4)
Cd1—N12	2.505 (2)	C1—C1^i^	1.498 (5)
S11—O11	1.444 (2)	C2—C3	1.387 (4)
S11—O12	1.448 (2)	C2—H2	0.9500
S11—N11	1.608 (2)	C3—C4	1.369 (4)
S11—C15	1.755 (3)	C3—C6	1.512 (4)
N11—C11	1.372 (3)	C4—C5	1.385 (4)
N12—C12	1.339 (3)	C4—H4	0.9500
N12—C11	1.358 (3)	C5—H5	0.9500
N13—C14	1.341 (3)	C6—H6A	0.9800
N13—C11	1.342 (3)	C6—H6B	0.9800
N14—C18	1.360 (3)	C6—H6C	0.9800
N14—H14A	0.948 (3)	N2—C7	1.328 (6)
N14—H14B	0.948 (3)	N2—C7D	1.377 (9)
C12—C13	1.381 (4)	N2—C8	1.418 (5)
C12—H12	0.9500	N2—C9	1.422 (4)
C13—C14	1.371 (4)	C8—H8A	0.9800
C13—H13	0.9500	C8—H8B	0.9800
C14—H14	0.9500	C8—H8C	0.9800
C15—C16	1.387 (4)	C9—H9A	0.9800
C15—C20	1.393 (4)	C9—H9B	0.9800
C16—C17	1.380 (4)	C9—H9C	0.9800
C16—H16	0.9500	C7—O1	1.274 (9)
C17—C18	1.403 (4)	C7—H7	0.9500
C17—H17	0.9500	C7D—O1D	1.101 (11)
C18—C19	1.411 (4)	C7D—H7'	0.9500
C19—C20	1.378 (4)		
			
N11^i^—Cd1—N11	116.97 (11)	C20—C19—C18	121.0 (2)
N11^i^—Cd1—N1^i^	102.88 (8)	C20—C19—H19	119.5
N11—Cd1—N1^i^	128.97 (8)	C18—C19—H19	119.5
N11^i^—Cd1—N1	128.97 (8)	C19—C20—C15	119.9 (2)
N11—Cd1—N1	102.88 (8)	C19—C20—H20	120.0
N1^i^—Cd1—N1	70.87 (11)	C15—C20—H20	120.0
N11^i^—Cd1—N12^i^	56.10 (7)	C5—N1—C1	118.2 (2)
N11—Cd1—N12^i^	95.54 (7)	C5—N1—Cd1	123.03 (19)
N1^i^—Cd1—N12^i^	134.18 (8)	C1—N1—Cd1	118.35 (17)
N1—Cd1—N12^i^	91.26 (7)	N1—C1—C2	121.2 (2)
N11^i^—Cd1—N12	95.54 (7)	N1—C1—C1^i^	115.69 (15)
N11—Cd1—N12	56.10 (7)	C2—C1—C1^i^	123.14 (15)
N1^i^—Cd1—N12	91.26 (7)	C3—C2—C1	120.2 (2)
N1—Cd1—N12	134.18 (8)	C3—C2—H2	119.9
N12^i^—Cd1—N12	127.65 (9)	C1—C2—H2	119.9
O11—S11—O12	115.91 (12)	C4—C3—C2	117.8 (3)
O11—S11—N11	112.65 (12)	C4—C3—C6	121.1 (2)
O12—S11—N11	104.99 (11)	C2—C3—C6	121.1 (3)
O11—S11—C15	107.39 (12)	C3—C4—C5	119.7 (3)
O12—S11—C15	108.56 (11)	C3—C4—H4	120.1
N11—S11—C15	106.97 (11)	C5—C4—H4	120.1
C11—N11—S11	122.04 (18)	N1—C5—C4	122.9 (3)
C11—N11—Cd1	101.86 (15)	N1—C5—H5	118.5
S11—N11—Cd1	134.42 (12)	C4—C5—H5	118.5
C12—N12—C11	117.0 (2)	C3—C6—H6A	109.5
C12—N12—Cd1	151.11 (19)	C3—C6—H6B	109.5
C11—N12—Cd1	90.87 (14)	H6A—C6—H6B	109.5
C14—N13—C11	114.8 (2)	C3—C6—H6C	109.5
C18—N14—H14A	126.8 (19)	H6A—C6—H6C	109.5
C18—N14—H14B	130 (2)	H6B—C6—H6C	109.5
H14A—N14—H14B	103 (2)	C7—N2—C7D	29.0 (4)
N13—C11—N12	125.7 (2)	C7—N2—C8	108.8 (4)
N13—C11—N11	123.4 (2)	C7D—N2—C8	136.6 (5)
N12—C11—N11	110.9 (2)	C7—N2—C9	133.8 (4)
N12—C12—C13	121.4 (3)	C7D—N2—C9	105.2 (5)
N12—C12—H12	119.3	C8—N2—C9	117.4 (3)
C13—C12—H12	119.3	N2—C8—H8A	109.5
C14—C13—C12	116.9 (2)	N2—C8—H8B	109.5
C14—C13—H13	121.5	H8A—C8—H8B	109.5
C12—C13—H13	121.5	N2—C8—H8C	109.5
N13—C14—C13	124.1 (3)	H8A—C8—H8C	109.5
N13—C14—H14	117.9	H8B—C8—H8C	109.5
C13—C14—H14	117.9	N2—C9—H9A	109.5
C16—C15—C20	120.0 (2)	N2—C9—H9B	109.5
C16—C15—S11	120.7 (2)	H9A—C9—H9B	109.5
C20—C15—S11	119.26 (19)	N2—C9—H9C	109.5
C17—C16—C15	120.1 (2)	H9A—C9—H9C	109.5
C17—C16—H16	119.9	H9B—C9—H9C	109.5
C15—C16—H16	119.9	O1—C7—N2	118.4 (6)
C16—C17—C18	121.0 (2)	O1—C7—H7	120.8
C16—C17—H17	119.5	N2—C7—H7	120.8
C18—C17—H17	119.5	O1D—C7D—N2	130.8 (10)
N14—C18—C17	122.0 (2)	O1D—C7D—H7'	114.6
N14—C18—C19	120.1 (3)	N2—C7D—H7'	114.6
C17—C18—C19	117.9 (2)		
			
O11—S11—N11—C11	64.3 (2)	N11—S11—C15—C16	124.8 (2)
O12—S11—N11—C11	−168.7 (2)	O11—S11—C15—C20	−175.67 (19)
C15—S11—N11—C11	−53.5 (2)	O12—S11—C15—C20	58.3 (2)
O11—S11—N11—Cd1	−133.41 (16)	N11—S11—C15—C20	−54.5 (2)
O12—S11—N11—Cd1	−6.41 (19)	C20—C15—C16—C17	−1.6 (4)
C15—S11—N11—Cd1	108.82 (17)	S11—C15—C16—C17	179.10 (19)
N11^i^—Cd1—N11—C11	74.58 (14)	C15—C16—C17—C18	0.6 (4)
N1^i^—Cd1—N11—C11	−62.58 (18)	C16—C17—C18—N14	−178.7 (2)
N1—Cd1—N11—C11	−138.26 (15)	C16—C17—C18—C19	0.9 (4)
N12^i^—Cd1—N11—C11	129.18 (15)	N14—C18—C19—C20	178.2 (2)
N12—Cd1—N11—C11	−3.22 (13)	C17—C18—C19—C20	−1.5 (4)
N11^i^—Cd1—N11—S11	−90.16 (16)	C18—C19—C20—C15	0.4 (4)
N1^i^—Cd1—N11—S11	132.68 (15)	C16—C15—C20—C19	1.1 (4)
N1—Cd1—N11—S11	57.01 (18)	S11—C15—C20—C19	−179.6 (2)
N12^i^—Cd1—N11—S11	−35.56 (17)	N11^i^—Cd1—N1—C5	93.2 (2)
N12—Cd1—N11—S11	−168.0 (2)	N11—Cd1—N1—C5	−48.3 (2)
N11^i^—Cd1—N12—C12	49.8 (4)	N1^i^—Cd1—N1—C5	−175.5 (3)
N11—Cd1—N12—C12	168.7 (4)	N12^i^—Cd1—N1—C5	47.6 (2)
N1^i^—Cd1—N12—C12	−53.3 (4)	N12—Cd1—N1—C5	−103.2 (2)
N1—Cd1—N12—C12	−117.5 (4)	N11^i^—Cd1—N1—C1	−94.7 (2)
N12^i^—Cd1—N12—C12	100.5 (4)	N11—Cd1—N1—C1	123.7 (2)
N11^i^—Cd1—N12—C11	−115.75 (15)	N1^i^—Cd1—N1—C1	−3.43 (14)
N11—Cd1—N12—C11	3.18 (13)	N12^i^—Cd1—N1—C1	−140.3 (2)
N1^i^—Cd1—N12—C11	141.18 (15)	N12—Cd1—N1—C1	68.8 (2)
N1—Cd1—N12—C11	77.01 (17)	C5—N1—C1—C2	2.5 (4)
N12^i^—Cd1—N12—C11	−65.01 (13)	Cd1—N1—C1—C2	−169.96 (19)
C14—N13—C11—N12	−1.9 (4)	C5—N1—C1—C1^i^	−178.4 (3)
C14—N13—C11—N11	176.8 (2)	Cd1—N1—C1—C1^i^	9.1 (4)
C12—N12—C11—N13	2.0 (4)	N1—C1—C2—C3	−1.5 (4)
Cd1—N12—C11—N13	174.2 (2)	C1^i^—C1—C2—C3	179.5 (3)
C12—N12—C11—N11	−176.8 (2)	C1—C2—C3—C4	−0.8 (4)
Cd1—N12—C11—N11	−4.64 (19)	C1—C2—C3—C6	178.1 (2)
S11—N11—C11—N13	−6.4 (3)	C2—C3—C4—C5	1.9 (4)
Cd1—N11—C11—N13	−173.6 (2)	C6—C3—C4—C5	−177.0 (3)
S11—N11—C11—N12	172.46 (17)	C1—N1—C5—C4	−1.3 (5)
Cd1—N11—C11—N12	5.3 (2)	Cd1—N1—C5—C4	170.8 (2)
C11—N12—C12—C13	−0.1 (4)	C3—C4—C5—N1	−1.0 (5)
Cd1—N12—C12—C13	−163.8 (3)	C7D—N2—C7—O1	8.8 (9)
N12—C12—C13—C14	−1.6 (4)	C8—N2—C7—O1	174.5 (5)
C11—N13—C14—C13	−0.1 (4)	C9—N2—C7—O1	−3.4 (8)
C12—C13—C14—N13	1.7 (4)	C7—N2—C7D—O1D	24.3 (8)
O11—S11—C15—C16	3.6 (2)	C8—N2—C7D—O1D	4.4 (16)
O12—S11—C15—C16	−122.4 (2)	C9—N2—C7D—O1D	−164.8 (10)

Symmetry code: (i) −*x*+1, *y*, −*z*+1/2.

### Hydrogen-bond geometry (Å, º)

**Table:**

*D*—H···*A*	*D*—H	H···*A*	*D*···*A*	*D*—H···*A*
N14—H14*B*···O1*D*^ii^	0.95 (1)	2.10 (1)	3.034 (7)	169 (3)
N14—H14*B*···O1^ii^	0.95 (1)	1.99 (2)	2.853 (5)	151 (3)
N14—H14*A*···O11^iii^	0.95 (1)	2.00 (1)	2.950 (3)	176 (3)
C12—H12···O12^iv^	0.95	2.48	3.393 (3)	162
C6—H6*C*···O1^v^	0.98	2.58	3.487 (5)	154
C6—H6*A*···N13^vi^	0.98	2.63	3.558 (4)	159
C8—H8*A*···O11^vii^	0.98	2.55	3.497 (4)	161

Symmetry codes: (ii) −*x*+1, −*y*, −*z*+1; (iii) −*x*+1/2, *y*−1/2, −*z*+1/2; (iv) *x*+1/2, −*y*+1/2, *z*+1/2; (v) −*x*+1, −*y*+1, −*z*+1; (vi) −*x*+1/2, *y*+1/2, −*z*+1/2; (vii) −*x*+1/2, −*y*+1/2, −*z*+1.
